# Bis(isobutyl­ammonium) phthalate monohydrate

**DOI:** 10.1107/S1600536808015201

**Published:** 2008-05-24

**Authors:** Zakaria Norzalida, Azhar Ariffin, Seik Weng Ng

**Affiliations:** a Department of Chemistry, University of Malaya, 50603 Kuala Lumpur, Malaysia

## Abstract

*N*-Isobutyl­phthalimic acid hydrolyzes to the title salt, 2C_4_H_12_N^+^·C_8_H_4_O_4_^−^·H_2_O, which adopts a hydrogen-bonded layer structure. In the anion, the carboxyl­ate groups are twisted with respect to the benzene ring [dihedral angles = 43.8 (1) and 50.9 (1)°].

## Related literature

For kinetic studies relating to the hydrolysis of *N*-isobutyl­phthalimic acid, see: Ariffin & Khan (2005 ▶); Khan & Ariffin (2003 ▶).
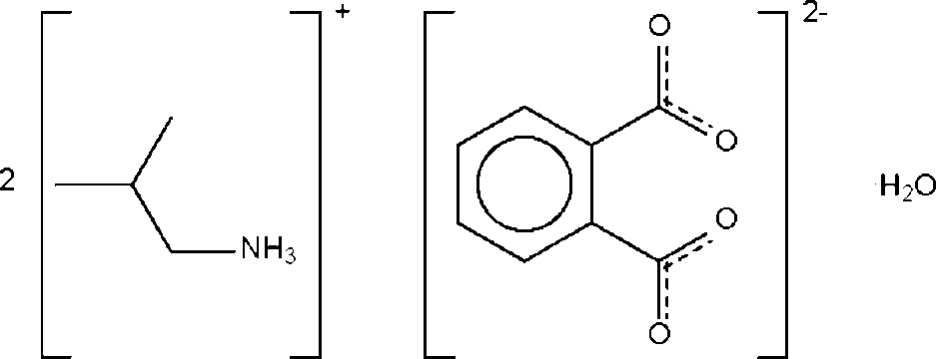


## Experimental

### 

#### Crystal data

2C_4_H_12_N^+^·C_8_H_4_O_4_^2−^·H_2_O
*M* *_r_* = 330.42
Triclinic, 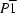

*a* = 8.8647 (4) Å
*b* = 9.4340 (5) Å
*c* = 12.9119 (6) Å
α = 72.298 (3)°
β = 79.449 (3)°
γ = 69.059 (3)°
*V* = 957.37 (8) Å^3^
*Z* = 2
Mo *K*α radiation
μ = 0.08 mm^−1^
*T* = 100 (2) K
0.32 × 0.08 × 0.08 mm

#### Data collection

Bruker SMART APEX diffractometer
Absorption correction: none
8057 measured reflections
4343 independent reflections
2454 reflections with *I* > 2σ(*I*)
*R*_int_ = 0.050

#### Refinement

*R*[*F* ^2^ > 2σ(*F* ^2^)] = 0.061
*wR*(*F* ^2^) = 0.156
*S* = 0.97
4343 reflections
241 parameters
15 restraints
H atoms treated by a mixture of independent and constrained refinement
Δρ_max_ = 0.33 e Å^−3^
Δρ_min_ = −0.27 e Å^−3^

### 

Data collection: *APEX2* (Bruker, 2007 ▶); cell refinement: *SAINT* (Bruker, 2007 ▶); data reduction: *SAINT*; program(s) used to solve structure: *SHELXS97* (Sheldrick, 2008 ▶); program(s) used to refine structure: *SHELXL97* (Sheldrick, 2008 ▶); molecular graphics: *X-SEED* (Barbour, 2001 ▶); software used to prepare material for publication: *publCIF* (Westrip, 2008 ▶).

## Supplementary Material

Crystal structure: contains datablocks global, I. DOI: 10.1107/S1600536808015201/lh2629sup1.cif

Structure factors: contains datablocks I. DOI: 10.1107/S1600536808015201/lh2629Isup2.hkl

Additional supplementary materials:  crystallographic information; 3D view; checkCIF report

## Figures and Tables

**Table 1:** Hydrogen-bond geometry (Å, °)

*D*—H⋯*A*	*D*—H	H⋯*A*	*D*⋯*A*	*D*—H⋯*A*
O1*w*—H1*w*1⋯O2	0.86 (1)	1.97 (2)	2.780 (2)	156 (3)
O1*w*—H1*w*2⋯O4^i^	0.86 (1)	1.97 (2)	2.780 (2)	157 (3)
N1—H1*n*1⋯O1	0.85 (1)	1.94 (1)	2.788 (2)	172 (2)
N1—H1*n*2⋯O3^ii^	0.87 (1)	1.91 (1)	2.755 (2)	167 (2)
N1—H1*n*3⋯O1*w*^iii^	0.86 (1)	1.99 (1)	2.823 (3)	164 (2)
N2—H2*n*1⋯O2	0.86 (1)	2.35 (2)	2.996 (2)	132 (2)
N2—H2*n*1⋯O4^i^	0.86 (1)	2.42 (2)	2.995 (3)	124 (2)
N2—H2*n*2⋯O2^i^	0.87 (1)	1.91 (1)	2.781 (2)	172 (2)
N2—H2*n*3⋯O3	0.87 (1)	1.89 (1)	2.741 (2)	166 (2)

Symmetry codes: (i) 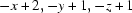; (ii) 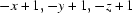; (iii) 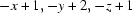.

**Fig. 1.**
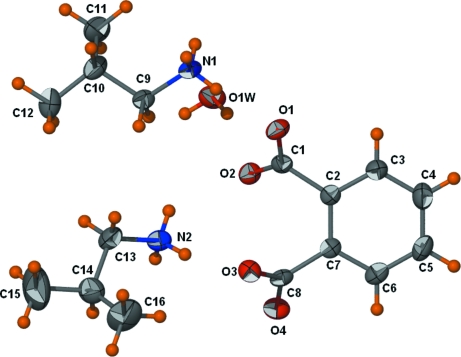
Thermal ellipsoid plot (Barbour, 2001) of [C4H12N]2[C8H4O4].H2O at the 70% probability level. Hydrogen atoms are drawn as spheres of arbitrary radiius.

## supplementary crystallographic information

### Comment

The title salt (Fig. 1) was obtained as a wet crystalline compound when *N*-isobutylphthalimic acid was left aside for several years. The acid has been shown by kinetic studies to be converted to phthalic acid and isobutylamine under neutral and acidic conditions (Ariffin & Khan, 2005; Khan Ariffin, 2003). In the anion, the carboxyl –CO_2_ groups are twisted with respect to the phenylene ring [dihedral angles 43.8 (1) and 50.9 (1) °]. Hydrogen bonds which involve the ammonium cations and water molecules link the components of the salt into a layer motif (Table 1).

### Experimental

*N*-Isobutylphthalimidic acid was synthesized as described earlier (Ariffin & Khan, 2005; Khan & Ariffin, 2003). The crystalline compound was left aside for several years. The hydrolyzed title salt was obtained as a wet crystalline compound.

### Refinement

Carbon-bound H-atoms were placed in calculated positions (C—H 0.95 to 0.99 Å) and were included in the refinement in the riding model approximation, with *U*(H) set to 1.2–1.5*U*(C). The oxygen- and nitrogen-bound H-atoms were located in a difference Fourier map, and were refined with restraints of O–H = N–H = 0.85±0.01 Å; H···H = 1.39±0.01 Å; their temperature factors were freely refined.

### Crystal data

**Table:**

2C_4_H_12_N^+^·C_8_H_4_O_4_^2–^·H_2_O	*Z* = 2
*M**_r_* = 330.42	*F*_000_ = 360
Triclinic, *P*1	*D*_x_ = 1.146 Mg m^−^^3^
Hall symbol: -P 1	Mo *K*α radiation λ = 0.71073 Å
*a* = 8.8647 (4) Å	Cell parameters from 941 reflections
*b* = 9.4340 (5) Å	θ = 2.5–22.7º
*c* = 12.9119 (6) Å	µ = 0.08 mm^−^^1^
α = 72.298 (3)º	*T* = 100 (2) K
β = 79.449 (3)º	Prism, colorless
γ = 69.059 (3)º	0.32 × 0.08 × 0.08 mm
*V* = 957.37 (8) Å^3^	

### Data collection

**Table:**

Bruker SMART APEX diffractometer	2454 reflections with *I* > 2σ(*I*)
Radiation source: fine-focus sealed tube	*R*_int_ = 0.050
Monochromator: graphite	θ_max_ = 27.5º
*T* = 100(2) K	θ_min_ = 2.5º
ω scans	*h* = −11→11
Absorption correction: None	*k* = −10→12
8057 measured reflections	*l* = −16→16
4343 independent reflections	

### Refinement

**Table:**

Refinement on *F*^2^	Secondary atom site location: difference Fourier map
Least-squares matrix: full	Hydrogen site location: inferred from neighbouring sites
*R*[*F*^2^ > 2σ(*F*^2^)] = 0.061	H atoms treated by a mixture of independent and constrained refinement
*wR*(*F*^2^) = 0.156	*w* = 1/[σ^2^(*F*_o_^2^) + (0.0703*P*)^2^] where *P* = (*F*_o_^2^ + 2*F*_c_^2^)/3
*S* = 0.97	(Δ/σ)_max_ = 0.001
4343 reflections	Δρ_max_ = 0.33 e Å^−^^3^
241 parameters	Δρ_min_ = −0.27 e Å^−^^3^
15 restraints	Extinction correction: SHELXL97 (Sheldrick, 2008), Fc^*^=kFc[1+0.001xFc^2^λ^3^/sin(2θ)]^-1/4^
Primary atom site location: structure-invariant direct methods	Extinction coefficient: 0.049 (5)

### Fractional atomic coordinates and isotropic or equivalent isotropic displacement parameters (Å^2^)

**Table:**

	*x*	*y*	*z*	*U*_iso_*/*U*_eq_	
O1	0.49468 (17)	0.58257 (19)	0.62388 (12)	0.0271 (4)	
O2	0.75147 (17)	0.58034 (18)	0.58411 (12)	0.0250 (4)	
O3	0.86510 (16)	0.23828 (19)	0.58460 (12)	0.0254 (4)	
O4	1.08162 (16)	0.21636 (19)	0.66133 (12)	0.0285 (4)	
O1w	0.7147 (2)	0.8989 (2)	0.50641 (15)	0.0342 (4)	
H1w1	0.696 (3)	0.811 (2)	0.535 (2)	0.078 (12)*	
H1w2	0.785 (3)	0.887 (3)	0.4520 (18)	0.096 (14)*	
N1	0.4142 (2)	0.8164 (2)	0.43079 (16)	0.0230 (5)	
H1n1	0.448 (3)	0.7460 (19)	0.4886 (14)	0.062 (10)*	
H1n2	0.3266 (18)	0.809 (2)	0.4159 (16)	0.034 (7)*	
H1n3	0.391 (2)	0.9076 (13)	0.4421 (17)	0.028 (7)*	
N2	0.9312 (2)	0.4473 (3)	0.39672 (16)	0.0259 (5)	
H2n1	0.873 (2)	0.5321 (15)	0.4154 (19)	0.054 (9)*	
H2n2	1.0323 (12)	0.440 (2)	0.3961 (17)	0.042 (8)*	
H2n3	0.917 (2)	0.3689 (16)	0.4499 (14)	0.037 (8)*	
C1	0.6391 (3)	0.5332 (3)	0.64401 (17)	0.0216 (5)	
C2	0.6832 (2)	0.4119 (3)	0.75120 (17)	0.0212 (5)	
C3	0.5823 (3)	0.4347 (3)	0.84480 (18)	0.0265 (5)	
H3	0.4850	0.5215	0.8393	0.032*	
C4	0.6217 (3)	0.3327 (3)	0.94606 (19)	0.0338 (6)	
H4	0.5509	0.3489	1.0092	0.041*	
C5	0.7644 (3)	0.2070 (3)	0.95501 (19)	0.0326 (6)	
H5	0.7928	0.1378	1.0244	0.039*	
C6	0.8653 (3)	0.1828 (3)	0.86233 (18)	0.0268 (6)	
H6	0.9630	0.0964	0.8688	0.032*	
C7	0.8264 (2)	0.2824 (3)	0.76022 (17)	0.0207 (5)	
C8	0.9328 (2)	0.2442 (3)	0.66050 (18)	0.0217 (5)	
C9	0.5386 (2)	0.7900 (3)	0.33858 (17)	0.0231 (5)	
H9A	0.5535	0.6865	0.3273	0.028*	
H9B	0.6430	0.7861	0.3584	0.028*	
C10	0.4971 (3)	0.9152 (3)	0.23207 (19)	0.0309 (6)	
H10	0.3883	0.9236	0.2148	0.037*	
C11	0.4909 (3)	1.0755 (3)	0.2390 (2)	0.0464 (8)	
H11A	0.4091	1.1074	0.2970	0.070*	
H11B	0.4626	1.1529	0.1692	0.070*	
H11C	0.5972	1.0693	0.2553	0.070*	
C12	0.6223 (3)	0.8638 (3)	0.14157 (19)	0.0369 (7)	
H12A	0.6251	0.7605	0.1383	0.055*	
H12B	0.7293	0.8575	0.1565	0.055*	
H12C	0.5931	0.9405	0.0716	0.055*	
C13	0.8993 (3)	0.4525 (3)	0.28710 (19)	0.0308 (6)	
H13A	0.9174	0.5469	0.2340	0.037*	
H13B	0.7845	0.4611	0.2877	0.037*	
C14	1.0072 (3)	0.3079 (3)	0.2514 (2)	0.0397 (7)	
H14	1.1214	0.2921	0.2627	0.048*	
C15	0.9981 (5)	0.3334 (5)	0.1306 (3)	0.0870 (13)	
H15A	1.0264	0.4277	0.0893	0.130*	
H15B	0.8878	0.3469	0.1174	0.130*	
H15C	1.0743	0.2421	0.1069	0.130*	
C16	0.9674 (5)	0.1631 (4)	0.3167 (3)	0.0663 (10)	
H16A	0.9732	0.1493	0.3944	0.100*	
H16B	1.0452	0.0715	0.2941	0.100*	
H16C	0.8576	0.1736	0.3043	0.100*	

### Atomic displacement parameters (Å^2^)

**Table:**

	*U*^11^	*U*^22^	*U*^33^	*U*^12^	*U*^13^	*U*^23^
O1	0.0211 (8)	0.0247 (10)	0.0298 (9)	−0.0054 (7)	−0.0062 (7)	0.0010 (7)
O2	0.0246 (8)	0.0226 (9)	0.0271 (8)	−0.0104 (7)	−0.0012 (7)	−0.0027 (7)
O3	0.0224 (8)	0.0283 (10)	0.0282 (9)	−0.0094 (7)	−0.0021 (7)	−0.0094 (7)
O4	0.0175 (8)	0.0308 (10)	0.0379 (10)	−0.0074 (7)	−0.0028 (7)	−0.0102 (8)
O1w	0.0366 (10)	0.0226 (11)	0.0420 (11)	−0.0109 (8)	0.0042 (9)	−0.0093 (9)
N1	0.0209 (10)	0.0173 (12)	0.0286 (12)	−0.0047 (8)	−0.0045 (9)	−0.0031 (10)
N2	0.0240 (10)	0.0219 (12)	0.0325 (12)	−0.0080 (9)	−0.0040 (9)	−0.0063 (10)
C1	0.0250 (11)	0.0155 (12)	0.0251 (12)	−0.0066 (10)	−0.0013 (10)	−0.0068 (10)
C2	0.0227 (11)	0.0195 (13)	0.0227 (12)	−0.0086 (10)	−0.0027 (9)	−0.0047 (10)
C3	0.0275 (12)	0.0233 (14)	0.0256 (13)	−0.0045 (10)	−0.0033 (10)	−0.0060 (10)
C4	0.0405 (14)	0.0363 (17)	0.0227 (13)	−0.0132 (12)	0.0010 (11)	−0.0059 (12)
C5	0.0423 (14)	0.0301 (15)	0.0227 (13)	−0.0113 (12)	−0.0097 (11)	0.0005 (11)
C6	0.0286 (12)	0.0230 (14)	0.0292 (13)	−0.0081 (10)	−0.0092 (10)	−0.0034 (11)
C7	0.0203 (11)	0.0199 (13)	0.0235 (12)	−0.0093 (10)	−0.0032 (9)	−0.0039 (10)
C8	0.0200 (11)	0.0136 (12)	0.0295 (13)	−0.0051 (9)	−0.0045 (9)	−0.0015 (10)
C9	0.0207 (11)	0.0201 (13)	0.0270 (12)	−0.0065 (9)	−0.0023 (9)	−0.0038 (10)
C10	0.0253 (12)	0.0320 (15)	0.0301 (13)	−0.0098 (11)	−0.0068 (10)	0.0027 (11)
C11	0.0577 (17)	0.0253 (16)	0.0409 (16)	−0.0109 (13)	0.0083 (14)	0.0027 (13)
C12	0.0443 (15)	0.0433 (18)	0.0267 (13)	−0.0213 (13)	−0.0027 (12)	−0.0059 (12)
C13	0.0396 (14)	0.0233 (14)	0.0286 (13)	−0.0081 (11)	−0.0115 (11)	−0.0029 (11)
C14	0.0430 (15)	0.0404 (18)	0.0359 (15)	−0.0071 (13)	−0.0066 (12)	−0.0156 (13)
C15	0.139 (4)	0.091 (3)	0.0396 (19)	−0.038 (3)	−0.001 (2)	−0.031 (2)
C16	0.116 (3)	0.0258 (18)	0.060 (2)	−0.0150 (18)	−0.023 (2)	−0.0156 (16)

### Geometric parameters (Å, °)

**Table:**

O1—C1	1.243 (2)	C7—C8	1.507 (3)
O2—C1	1.270 (2)	C9—C10	1.519 (3)
O3—C8	1.263 (3)	C9—H9A	0.9900
O4—C8	1.251 (2)	C9—H9B	0.9900
O1w—H1w1	0.861 (10)	C10—C12	1.522 (3)
O1w—H1w2	0.858 (10)	C10—C11	1.523 (4)
N1—C9	1.483 (3)	C10—H10	1.0000
N1—H1n1	0.852 (9)	C11—H11A	0.9800
N1—H1n2	0.865 (9)	C11—H11B	0.9800
N1—H1n3	0.861 (9)	C11—H11C	0.9800
N2—C13	1.477 (3)	C12—H12A	0.9800
N2—H2n1	0.860 (9)	C12—H12B	0.9800
N2—H2n2	0.874 (9)	C12—H12C	0.9800
N2—H2n3	0.873 (9)	C13—C14	1.508 (4)
C1—C2	1.514 (3)	C13—H13A	0.9900
C2—C3	1.389 (3)	C13—H13B	0.9900
C2—C7	1.406 (3)	C14—C16	1.496 (4)
C3—C4	1.386 (3)	C14—C15	1.518 (4)
C3—H3	0.9500	C14—H14	1.0000
C4—C5	1.384 (3)	C15—H15A	0.9800
C4—H4	0.9500	C15—H15B	0.9800
C5—C6	1.383 (3)	C15—H15C	0.9800
C5—H5	0.9500	C16—H16A	0.9800
C6—C7	1.386 (3)	C16—H16B	0.9800
C6—H6	0.9500	C16—H16C	0.9800
			
H1w1—O1w—H1w2	107.2 (15)	C9—C10—C12	108.7 (2)
C9—N1—H1n1	109.2 (17)	C9—C10—C11	112.0 (2)
C9—N1—H1n2	108.1 (14)	C12—C10—C11	110.4 (2)
H1n1—N1—H1n2	109.4 (13)	C9—C10—H10	108.5
C9—N1—H1n3	113.4 (15)	C12—C10—H10	108.5
H1n1—N1—H1n3	108.9 (13)	C11—C10—H10	108.5
H1n2—N1—H1n3	107.9 (12)	C10—C11—H11A	109.5
C13—N2—H2n1	111.6 (16)	C10—C11—H11B	109.5
C13—N2—H2n2	109.3 (15)	H11A—C11—H11B	109.5
H2n1—N2—H2n2	106.4 (13)	C10—C11—H11C	109.5
C13—N2—H2n3	116.3 (15)	H11A—C11—H11C	109.5
H2n1—N2—H2n3	107.0 (13)	H11B—C11—H11C	109.5
H2n2—N2—H2n3	105.7 (13)	C10—C12—H12A	109.5
O1—C1—O2	125.4 (2)	C10—C12—H12B	109.5
O1—C1—C2	117.22 (19)	H12A—C12—H12B	109.5
O2—C1—C2	117.30 (18)	C10—C12—H12C	109.5
C3—C2—C7	119.0 (2)	H12A—C12—H12C	109.5
C3—C2—C1	118.49 (19)	H12B—C12—H12C	109.5
C7—C2—C1	122.38 (19)	N2—C13—C14	111.78 (19)
C4—C3—C2	121.0 (2)	N2—C13—H13A	109.3
C4—C3—H3	119.5	C14—C13—H13A	109.3
C2—C3—H3	119.5	N2—C13—H13B	109.3
C5—C4—C3	119.9 (2)	C14—C13—H13B	109.3
C5—C4—H4	120.1	H13A—C13—H13B	107.9
C3—C4—H4	120.1	C16—C14—C13	112.4 (2)
C6—C5—C4	119.6 (2)	C16—C14—C15	110.8 (3)
C6—C5—H5	120.2	C13—C14—C15	110.0 (3)
C4—C5—H5	120.2	C16—C14—H14	107.8
C5—C6—C7	121.2 (2)	C13—C14—H14	107.8
C5—C6—H6	119.4	C15—C14—H14	107.8
C7—C6—H6	119.4	C14—C15—H15A	109.5
C6—C7—C2	119.3 (2)	C14—C15—H15B	109.5
C6—C7—C8	119.4 (2)	H15A—C15—H15B	109.5
C2—C7—C8	121.22 (19)	C14—C15—H15C	109.5
O4—C8—O3	125.5 (2)	H15A—C15—H15C	109.5
O4—C8—C7	117.10 (19)	H15B—C15—H15C	109.5
O3—C8—C7	117.40 (18)	C14—C16—H16A	109.5
N1—C9—C10	113.95 (18)	C14—C16—H16B	109.5
N1—C9—H9A	108.8	H16A—C16—H16B	109.5
C10—C9—H9A	108.8	C14—C16—H16C	109.5
N1—C9—H9B	108.8	H16A—C16—H16C	109.5
C10—C9—H9B	108.8	H16B—C16—H16C	109.5
H9A—C9—H9B	107.7		
			
O1—C1—C2—C3	−43.9 (3)	C1—C2—C7—C6	174.98 (19)
O2—C1—C2—C3	133.5 (2)	C3—C2—C7—C8	174.7 (2)
O1—C1—C2—C7	139.7 (2)	C1—C2—C7—C8	−8.9 (3)
O2—C1—C2—C7	−42.9 (3)	C6—C7—C8—O4	−51.4 (3)
C7—C2—C3—C4	0.4 (3)	C2—C7—C8—O4	132.5 (2)
C1—C2—C3—C4	−176.1 (2)	C6—C7—C8—O3	126.5 (2)
C2—C3—C4—C5	0.8 (4)	C2—C7—C8—O3	−49.7 (3)
C3—C4—C5—C6	−1.1 (4)	N1—C9—C10—C12	−172.76 (19)
C4—C5—C6—C7	0.1 (4)	N1—C9—C10—C11	65.0 (3)
C5—C6—C7—C2	1.2 (3)	N2—C13—C14—C16	68.6 (3)
C5—C6—C7—C8	−175.0 (2)	N2—C13—C14—C15	−167.4 (2)
C3—C2—C7—C6	−1.4 (3)		

### Hydrogen-bond geometry (Å, °)

**Table:**

*D*—H···*A*	*D*—H	H···*A*	*D*···*A*	*D*—H···*A*
O1*w*—H1*w*1···O2	0.86 (1)	1.97 (2)	2.780 (2)	156 (3)
O1*w*—H1*w*2···O4^i^	0.86 (1)	1.97 (2)	2.780 (2)	157 (3)
N1—H1*n*1···O1	0.85 (1)	1.94 (1)	2.788 (2)	172 (2)
N1—H1*n*2···O3^ii^	0.87 (1)	1.91 (1)	2.755 (2)	167 (2)
N1—H1*n*3···O1*w*^iii^	0.86 (1)	1.99 (1)	2.823 (3)	164 (2)
N2—H2*n*1···O2	0.86 (1)	2.35 (2)	2.996 (2)	132 (2)
N2—H2*n*1···O4^i^	0.86 (1)	2.42 (2)	2.995 (3)	124 (2)
N2—H2*n*2···O2^i^	0.87 (1)	1.91 (1)	2.781 (2)	172 (2)
N2—H2*n*3···O3	0.87 (1)	1.89 (1)	2.741 (2)	166 (2)

Symmetry codes: (i) −*x*+2, −*y*+1, −*z*+1; (ii) −*x*+1, −*y*+1, −*z*+1; (iii) −*x*+1, −*y*+2, −*z*+1.
